# Impact of post-tuberculosis lung disease on health-related quality of life in patients from two tertiary hospitals in Lagos, Nigeria

**DOI:** 10.7196/AJTCCM.2021.v27i2.135

**Published:** 2021-06-23

**Authors:** O B Ozoh, O O Ojo, M G Dania, S K Dede, O A Adegboyega, N K Irurhe, M Olowoyeye, O O Adeyeye

**Affiliations:** 1 Department of Medicine, College of Medicine, University of Lagos, Nigeria; 2 Department of Medicine, Lagos University Teaching Hospital, Nigeria; 3 Department of Medicine, College of Medicine, Lagos State University, Nigeria; 4 Department of Medicine, Lagos State University Teaching Hospital, Nigeria; 5 Department of Radiodiagnosis, College of Medicine, Lagos State University, Nigeria; 6 Department of Radiodiagnosis, Lagos State University Teaching Hospital, Nigeria; 7 Department of Radiodiagnosis, College of Medicine, University of Lagos, Nigeria

**Keywords:** tuberculosis, post-TB, TB sequelae, health-related quality of life, chronic respiratory diseases

## Abstract

**Background:**

Post-tuberculosis (TB) lung disease is a recognised cause of chronic respiratory disease, and its impact on health-related quality of life (HRQoL) has not been extensively described.

**Objectives:**

To describe the clinical profile, spirometry impairment and impact of post-TB lung disease on HRQoL among patients attending two tertiary hospitals in Lagos, Nigeria.

**Methods:**

We conducted a cross-sectional study and obtained data through interviews, chart reviews and physical examination. We measured dyspnoea severity using the Medical Research Council (MRC) scale, HRQoL with the St George’s respiratory questionnaire (SGRQ) and performed spirometry. Univariate regression was used to explore the associations between selected variables and HRQoL.

**Results:**

A total of 59 participants were recruited and their median (interquartile range (IQR)) age was 45 (36 - 60) years. The most frequent symptom was cough (93.2%; n=55) and sputum production (91.5%; n=54). Less than two-thirds of the participants (62.4%; n=38) had received treatment for TB on more than one occasion, 50.8% (n=30) had moderate to very severe dyspnoea on the MRC scale and 88.7% (n=47/53) had abnormal spirometry with the mixed pattern predominating in 56.6% (n=30) of the participants. The mean (standard deviation (SD)) SGRQ component score for symptoms was 43.89 (18.66), followed by activity (42.50 (22.68)), impact (29.41 (17.82)) and total components (35.78 (17.25)). Dyspnoea, cough, sputum production and weight loss were associated with worsened HRQoL.

**Conclusion:**

Post-TB lung disease was characterised by a high symptom burden, severe spirometry impairment and poor HRQoL. There is a need for increased recognition and development of guidelines for diagnosis and treatment of post-TB lung disease and for further studies to explore preventive strategies.

## Background

The World Health Organization (WHO) estimates that chronic respiratory diseases (CRDs) would be responsible for the largest increase in global mortality in the coming decades.^[1]^ Previous history of pulmonary tuberculosis (PTB) is now recognised as an important risk factor for CRD along with traditional risk factors such as exposure to cigarette smoke and air pollution (ambient, household and occupational).^[2–4]^ Excess mortality risk after complete TB treatment has been reported, suggesting that microbiological cure, though an important public health priority, may be a form of reductionism and not a holistic outcome measure from the patient’s perspective.^[5]^

Estimates of the global burden of TB only calculate the incidence, prevalence, mortality and disability-adjusted life years (DALYs) lost due to active TB.^[6]^ There are no consideration for DALYs lost due to disability from TB sequelae, or reduced life expectancy in patients that are microbiologically cured despite compelling evidence from epidemiological studies and systematic reviews that a history of TB increases the risk of CRD.^[7]^ Physiological and anatomical changes in the lungs as a sequel of TB present clinically as lung function impairments, bronchiectasis and aspergillosis.^[3-4,7-9]^ Computed tomography (CT) scans of post-TB patients analysed in a systematic review revealed a high prevalence of residual bronchiectasis (86%), nodules (55%), emphysema (45%) and cavitation (19%).^[9]^ Abnormal spirometry has also been reported in up to 70% of symptomatic and 54% of asymptomatic participants post TB treatment.^[10]^

The burden of TB is high in sub-Saharan Africa (SSA) with 17 countries ranked among the 30 high burden countries globally in 2019. ^[11]^ About a quarter of global new TB cases are reported in Africa, and Nigeria tops the list in the 6th position among high burden countries.^[11]^ At a recent International Post-TB Symposium in 2019, a definition for post-TB lung disease was proposed as ‘evidence of chronic respiratory abnormality, with or without symptoms, attributable at least in part to previous tuberculosis’.^[13]^ Based on this definition, the burden of post-TB lung disease is likely to be high in SSA, but only a few studies have reported on this in Africa. Furthermore, there is a global paucity of data on the clinical profile, practice experience and the impact of post-TB lung disease on health-related quality of life (HRQoL).^[9,13-14]^ It is well recognised that disease burden transcends symptoms, and deterioration in quality of life as perceived by the patient is an important concept that has significant bearing on morbidity and mortality.^[15]^ Assessment of quality of life makes for better characterisation of the disease burden and supports the development of interventions, including guidelines. At present, most national and international guidelines, including the Nigerian National TB guidelines, do not single out post-TB lung disease as a potential adverse sequelae of TB treatment nor provide guidance on the diagnosis or treatment.^[14]^

The aim of this present study was to describe the clinical profile, spirometry pattern and HRQoL in patients being managed for postTB lung disease. We also sought to explore the associations between HRQoL and clinical variables.

## Methods

### Study design

This was a cross-sectional hospital-based study in patients with physician diagnosis of post-TB lung disease attending outpatient clinics from two tertiary care hospitals in Lagos, Nigeria. The study period was between October 2018 and October 2019.

### Ethics

The study was approved by the Institutional Review Board of the two tertiary institutions where the research was conducted (ref. nos ADM/ DCST/HREC/APP/2562 and LREC.06/10/1088). Written informed consent was obtained from all participants.

### Study population and participant recruitment

We recruited all consecutively consenting adults (18 years of age and older) who were already diagnosed and being managed for post-TB lung disease who met inclusion criteria. The prior physician diagnosis of post-TB lung disease had been based on the presence of respiratory symptoms and/or abnormal imaging following completion of TB treatment with a negative sputum GeneXpert test performed at the time of presentation. Participants with active TB (positive Gene Xpert) or those currently on TB treatment were excluded. We also excluded participants who were current smokers or ex-smokers with more than 10 packs per year of smoking. Recruitment of patients with acute exacerbation of illness, or who were otherwise unfit and unable to perform spirometry testing, was delayed until acute symptoms resolved. We did not perform spirometry for patients with persistent hemoptysis.

The present study was a pilot study to describe the clinical profile and burden of post-TB lung disease in patients attending our clinics over a 1-year period; therefore, sample size was not calculated.

### Data collection

Data were obtained by a trained interviewer (a doctor) using a standard proforma. This involved chart review of case notes, face-to-face interviews, and general physical examination of the participants. We documented sociodemographic information including history of tobacco smoking, dates and number of previous TB treatment, HIV status, presenting symptoms, comorbid medical conditions, and current medications. We obtained previous chest radiographs, and these were examined independently by two radiologists who then agreed on a final report. A third radiologist was consulted to resolve any disagreement in the reporting.

The level of dyspnoea was assessed using the Medical Research Council (MRC) dyspnoea scale. The MRC dyspnoea scale is a five-point scale that measures the level of breathlessness and is used to assess the severity of dyspnoea. It is categorised as follows: Grade 1 = short of breath with strenuous exercise; Grade 2 = short of breath when hurrying on a level or walking up a slight hill; Grade 3 = walks slower than people of the same age on the level or must stop for breath when walking at own pace; Grade 4 = stops for breath after 100 m at own pace; and Grade 5 = too breathless to leave the house.^[16]^

Respiratory impairment and HRQoL were assessed using the St George’s Respiratory questionnaire (SGRQ) that was administered by the interviewer.^[17]^ The SGRQ measures health status in patients with respiratory impairment and has been shown to correlate significantly with measures of disease activity such as cough, dyspnoea, lung function and other quality of life measures such as the Short Form 36 (SF36) health survey questionnaire.^[18]^ It has been found to be reliable in assessing quality of life in patients with airway diseases such as asthma, chronic obstructive pulmonary disease (COPD) and bronchiectasis, and has been used reliably to assess quality of life in patients with COPD in Nigeria.^[19]^ The SGRQ comprises a series of weighted responses that cut across three domains: symptoms, activity and impact (psycho-social). A total score is also computed, and the score ranges from 0 to 100 with high scores representing worsened quality of life.

Weight and height were measured by standard methods and body mass index (BMI) calculated in kg/m² . BMI was categorised as underweight (<18 kg/m² ), normal weight (18 - 24.5 kg/m² ), overweight (25 - 29 kg/m² ), and obese (≥30 kg/m² ). Oxygen saturation was measured on the middle finger for all participants using a pulse oximeter.

### Spirometry testing

Spirometry (pre- and post-bronchodilator) was performed by a trained doctor following a standard operating procedure designed according to the American Thoracic Society/European Respiratory Society (ATS/ERS) standards for spirometry performance.^[20]^

The spirometer was calibrated daily to check accuracy of the equipment and a minimum of three and maximum of eight acceptable and repeatable tests performed. Each test was independently assessed for quality by OBO who is trained in spirometry quality assurance and only tests that met the ATS/ERS standards for spirometry interpretation were documented.^[21]^ The forced expiratory volume in one second (FEV_1_ ), forced vital capacity (FVC) and the ratio of the FEV_1_ /FVC were measured. Post-bronchodilator spirometry was performed 20 minutes after administration of 400µg of inhaled salbutamol using a metered dose inhaler and a spacer. Significant bronchodilator response was an improvement in FEV_1_ or FVC of ≥12% and 200 mL post-bronchodilator.

We used the global lung function initiative (GLI) normative equation for ‘African Americans’ to determine the percentages of predicted values, the Z-scores and the spirometry pattern.^[22]^ The ERS recommends the use of the African American equation for black persons.^[23]^ Spirometry pattern was characterised as obstructive (FEV_1_ /FVC <lower limit of normal (LLN) with FVC ≥LLN), probable restrictive (FVC <LLN with FEV_1_ /FVC ≥LLN), mixed (obstructive and probable restrictive) (FEV_1_ /FVC <LLN with FVC <LLN) and normal (FEV_1_/FVC ≥LLN with FVC ≥LLN). Severity of the impairment in lung function for participants was graded using the Z-scores for the FEV_1_ as follows: mild (≥ −2.0), moderate (−2.5 - −2.0), moderately severe (−3.0 - −2.5), severe (−4.0 - −3.0), and very severe (< −4.0). This categorisation correlates with the ATS/ERS categorisation and overcomes biases related to age, height and sex. ^[24]^

### Data handling and statistical analysis

Summarised data were presented as counts (percentages), mean (standard deviation) or median (interquartile range (IQR). The scores on the SGRQ were computed using the Excel scoring calculator from the developer (Microsoft Corp., USA). The association between quality of life and selected variables was explored using univariate regression analysis. A *p*-value <0.05 was considered significant for all associations.

## Results

A total of 59 patients were recruited into the study from two tertiary hospitals in Lagos. There were 54.2% (n=32) females and 45.8% (n=27) males. Age range was 21 - 81 years, and the median (IQR) age was 45 (36 - 60) years. Sociodemographic characteristics of participants are shown in Table 1. A tenth of the participants (10.2%; n=6) were ex-tobacco smokers with less than 10 packs per year of smoking and 8.5% (n=5) were HIV-positive.

**Table 1:** Sociodemographic characteristics of participants (N=59)

**Variable**	***n *(%)**
**Age group, years**	
20 - 38	21 (35.6)
40 - 59	23 (39.0)
≥60	15 (24.5)
**Gender**	
Male	27 (35.8)
Female	32 (54.2)
**Marital status**	
Single	11 (18.6)
Married	44 (74.6)
Others	4 (6.8)
**Highest level of education**	
None	2 (3.4)
Primary	13 (22.0)
Secondary	24 (40.7)
Post-secondary	20 (33.9)

**Fig. 1:**
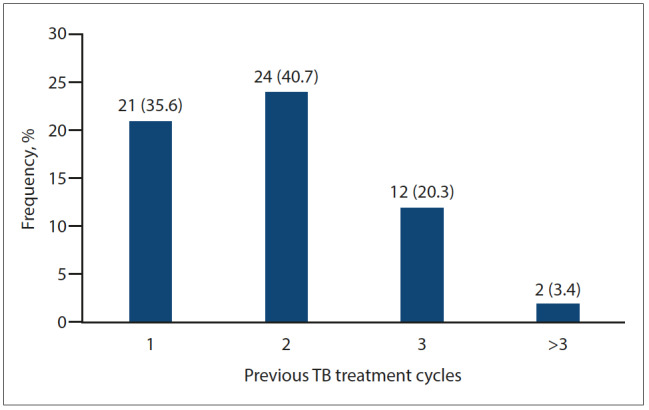
Number of cycles of previous tuberculosis treatment (n (%)).

Almost two-thirds of the participants (62.4%; n=38) completed treatment for TB on more than one occasion and 23.7% (n=14) had received treatment ≥3 times (Fig. 1). Based on self-report, TB was bacteriologically confirmed in 79.7% (n=47) of participants prior to first treatment and 42.1% (n=16/38) of those treated on more than one occasion. Most of the participants (91.5%; n=54) received their TB treatment at a secondary care hospital (general hospital) while 8.5% (n=5) received it at a primary healthcare facility. The median (IQR) duration between recruitment into this study and the first and most recent TB treatment was 10 (4 - 19) years and 4 (2 - 8) years, respectively.

### Clinical features

Cough and sputum production were the most common symptoms reported by 93.2% (n=55) and 91.5% (n=54) of the participants, respectively. The distribution of symptoms and signs among participants are shown in Table 2.

**Table 2:** Clinical features of participants with post-TB lung disease (N=59)

**Variable**	***n *(%)**
**Cough**	55 (93.2)
**Sputum production**	54 (91.5)
**Shortness of breath**	41 (69.5)
**Fatigue**	34 (57.6)
**Blood in sputum**	30 (50.8)
**Weight loss**	25 (42.4)
**Chest pain**	13 (22.0)
**Wheezing**	10 (16.9)
**Orthopnoea**	7 (11.9)
**Leg swelling**	6 (10.2)
**Foul-smelling sputum**	4 (6.8)
**Paroxysmal nocturnal dyspnea**	3 (5.1)
**Halitosis**	1 (1.7)
**BMI (kg/m²)**	
Underweight	18 (30.5)
Normal	32 (54.2)
Overweight	5 (8.5)
Obese	4 (6.8)
**Pallor**	6 (10.2)
**Digital clubbing**	18 (30.5)
**Pedal oedema**	2 (3.4)

**Fig. 2:**
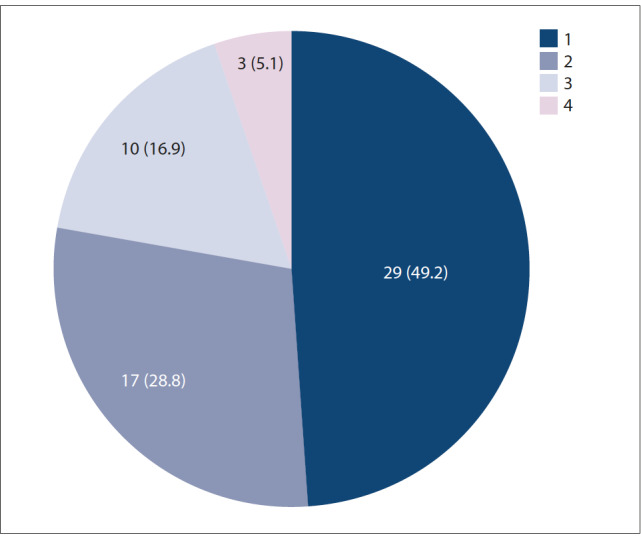
Grades of dyspnoea among 59 participants using the MRC dyspnoea scale (n (%)).

The mean (SD) BMI was 21.5 (4.1) kg/m² and mean (SD) oxygen saturation was 97% (1.07). MRC dyspnoea scale grading showed that about half of the participants had moderate to very severe dyspnoea (Fig. 2).

### Complications and current treatment

The frequency of radiographic abnormalities as documented in the imaging report on the clinical notes were as follows (multiple reports were documented in some cases): bronchiectasis (61%; n=36), destroyed lung (28.8%; n=17), aspergilloma (18.6%; n=11) and lung collapse (8.4%; n=5). COPD was documented for 18.6% (n=11) of the participants based on previous spirometry.

Less than half of the participants (45.8%; n=27) had comorbidities of which hypertension was the most common (18.6%; n=11), followed by cor pulmonale confirmed by echocardiography (5.1%; n=3).

Most of the participants (n=54) were taking medication for post-TB complications and 51/54 reported improvement in symptoms with the use of the prescribed medications. Current medications specifically for post-TB lung diseases were inhaled corticosteroid plus a long-acting bronchodilator (45.5%; n=27), azithromycin twice weekly (18.6%; n=11), itraconazole (16.9%; n=10) and oral steroids (1.7%; n=1).

### Spirometry findings

Spirometry was performed in 53/59 participants. Six participants had persistent haemoptysis that precluded spirometry. The mean spirometry values pre and post bronchodilator are shown in Table 3. A mixed pattern (obstructive and probable restrictive) was the most predominant abnormality both pre and post bronchodilator. Six participants had normal spirometry. Most of the participants (69.8; n=37) had severe to very severe impairment pre bronchodilator based on FEV_1_ Z-score (Table 3). The mean (SD) postbronchodilator reversibility in the FEV_1_ was 9.63 (17.85 L) and FVC was 3.66 (12.71 L). Participants had significant improvements in FEV_1_ (24.5%; n=13) and FVC (17.0%; n=9) following bronchodilation.

**Table 3:** Spirometry indices and pattern pre and post bronchodilator (N=53)

**Spirometry indices**	**Pre bronchodilator,** mean (SD)*	**Post bronchodilator,** mean (SD)*
**FVC, L**	1.93 (0.60)	1.97 (0.58)
**FVC Z-score**	–3.08 (1.85)	–2.97 (1.80)
**FVC % predicted, median (IQR)**	53.3 (40.8 - 72.9)	55.4 (41.4 - 69.3)
**FEV_1_, L**	1.22 (0.53)	1.29 (0.50)
**FEV_1_ Z-score**	–3.50 (1.82)	–3.36 (1.76)
**FEV_1_ % predicted, median (IQR)**	40.2 (29.0 - 55.3)	44.3 (32.6 - 57.4)
**FEV_1_/FVC%**	61.83 (15.42)	65.0 (15.68)
**FEV_1_/FVC Z-score, median (IQR) **	–2.67 (–3.79 - –1.06)	–2.39 (–3.30 - –0.78)
**FEV_1_ Z-score grading, n(%)**		
Mild (≥ −2.0)	10 (18.9)	10 (18.9)
Moderate (–2.5 - –2.0)	0	2 (3.8)
Moderately severe (−3.0 - −2.5)	6 (11.3)	6 (11.3)
Severe (−4.0 - −3.0)	12 (22.6)	14 (26.4)
Very severe (< −4.0)	25 (47.2)	21 (39.6)
**Spirometry pattern, n(%)**		
Mixed	30 (56.6)	25 (47.2)
Probable restriction	11 (20.8)	17 (32.1)
Obstructive	6 (11.3)	5 (9.4)
Normal	6 (11.3)	6 (11.3)

SD = standard deviation

IQR = interquartile range

FEV_1_ = forced expiratory volume in one second

FVC = forced vital capacity

* Unless otherwise specified

### Health-related quality of life using the SGRQ

The mean (SD) SGRQ component scores for all 59 participants were 43.89 (18.66), 42.50 (22.68), 29.41 (17.82) and 35.78 (17.25) in the symptoms, activity, impact, and total domains, respectively. There was no difference in mean (SD) scores between males (42.43 (19.0), 45.42 (26.08), 30.40 (18.80), and 36.95 (19.29)) and females (45.12 (18.59), 40.03 (19.45), 28.57 (17.22) and 34.79 (15.56)) in the symptoms, activity, impact, and total scores, respectively.

### Association between SGRQ scores and selected variables

The association between SGRQ component scores and selected clinical and spirometry variables was analysed for 53 participants who had spirometry data. Table 4 shows the univariate analysis with unadjusted odds ratio (OR) for the component scores on the SGRQ. Increasing dyspnoea, presence of cough, sputum production and weight loss were significantly associated with higher scores (poor quality of life) for all components of the SGRQ. There was no association between the number of cycles of TB treatment and quality of life scores. The small sample size precluded performance of multivariate analysis.

**Table 4:** Univariate regression analysis for association between SGRQ component scores and selected variables

**Variables**	**Symptoms score** **OR (*p*-value)**	**Activity score** **OR (*p*-value)**	**Impact score** **OR (*p*-value)**	**Total score** **OR (*p*-value)**
Age	0.006 (0.97)	0.07 (0.62)	–0.27 (0.05)	–0.12 (0.38)
Sex	0.10 (0.49)	–0.05 (0.75)	0.04 (0.80)	0.02 (0.88)
BMI	0.03 (0.81)	0.07 (0.62)	0.004 (0.98)	0.04 (0.80)
MRC dyspnoea scale	0.42 (0.002)	0.50 (<0.001)	0.39 (0.004)	0.49 (<0.001)
FVC-pre	–0.25 (0.07)	–0.35 (0.01)	–0.15 (0.29)	–0.26 (0.06)
FVC-post	–0.27 (0.05)	–0.36 (0.009)	–0.12 (0.40)	–0.25 (0.07)
FEV_1_-pre	–0.33 (0.02)	–0.45 (0.001)	–0.18 (0.19)	–0.33 (0.02)
FEV_1_-post	–0.37 (0.006)	–0.51 (<0.001)	–0.21 (0.13)	–0.38 (0.005)
FEV_1_/FVC-pre	–0.21 (0.12)	–0.31 (0.02)	–0.07 (0.60)	–0.20 (0.15)
FEV_1_/FVC-post	–0.28 (0.04)	–0.39 (0.004)	–0.18 (0.21)	–0.30 (0.03)
Cough	0.38 (0.005)	0.33 (0.02)	0.32 (0.02)	0.37 (0.006)
Sputum production	0.38 (0.005)	0.33 (0.02)	0.32 (0.02)	0.37 (0.006)
Fatigue	0.22 (0.11)	0.08 (0.55)	0.20 (0.14)	0.19 (0.18)
Blood in sputum	–0.07 (0.62)	–0.10 (0.47)	0.10 (0.49)	0.002 (0.99)
Weight loss	0.32 (0.02)	0.30 (0.03)	0.31 (0.02)	0.36 (0.01)
Number of cycles of TB treatment	0.13 (0.34)	0.09 (0.50)	–0.04 (0.76)	0.04 (0.79)

OR = odds ratio

BMI = body mass index

MRC = Medical Research Council

FVC = forced vital capacity

FEV_1_ = forced expiratory volume in 1 second

## Discussion

The impact of post-TB lung disease on HRQoL has not been extensively reported, more so, in the African context where the burden of TB is very high. The paucity of such data may lead to perceived underestimation of disease burden and could hinder prioritisation of an important public health challenge.

In this present study, we found high symptom burden and poor HRQoL across all components of the SGRQ, particularly in the symptoms and activities components. Increasing dyspnoea showed significant association with poor HRQoL across all component scores and post-bronchodilator FEV_1_ was also strongly associated with the symptom, activity, and total scores. Cough, sputum production, weight loss and increasing dyspnoea were also associated with poor HRQoL. Other notable findings from this present study were the reports of multiple courses of TB treatment and severe lung function impairment.

The SGRQ has been used extensively in patients with airway diseases such as COPD, asthma and bronchiectasis, and its utility has been expanded to other respiratory diseases including its validation for post-TB lung disease.^[25]^ The pervasiveness of bronchiectasis and COPD in post-TB lung disease underscores the reported validity as a reliable HRQoL tool in this condition. The HRQoL scores we have reported are above the highest mean score (about 15) in a normal healthy population indicating poor quality of life in these patients with post-TB lung disease.^[26]^

Few studies have assessed the clinical profile and quality of life in people with post-TB lung disease. A study by Pasipanodya *et al*.^[25]^ reported a 13-point increase (worsening QoL) in total SGRQ score after TB treatment. This was corroborated by a study by Daniels et al.^[27]^ who used a different HRQoL assessment tool, the Short Form 12 (SF-12) health survey questionnaire, and reported poor HRQoL post-TB in a South African population. Similar to our findings, these studies also report lower HRQoL in relation to the physical domain compared with the mental domain that was independent of gender. There was no association between HRQoL and age in this present study unlike in reports from patients with active TB.^[28]^ In a recent prospective study in Malawi^[29]^, HRQoL scores on the SGRQ improved significantly and progressively at 6 months and 12 months following treatment compared with baseline; however, the long-term outcomes among those who develop residual lung diseases are not known.

HRQoL is a multidimensional concept that encompasses physical, social, spiritual, and economic factors and provides a holistic understanding of disease burden. It provides guidance towards disease management by highlighting modifiable predictors of HRQoL that could improve overall outcome. Dyspnoea is a cross cutting predictor of HRQoL and interventions aimed at improving dyspnoea may be an important target for enhancing QoL. Interestingly, a pilot study conducted in Uganda^[30]^ showed that a short (6 weeks) pulmonary rehabilitation program reduced dyspnoea and improved exercise tolerance in post-TB patients. These preliminary benefits require substantiation in future research. Moreover, the self-reported improvements in symptoms following use of medications such as a bronchodilator plus inhaled steroid reported in the present study warrant further evaluation in future well-designed studies.

Poor lung function is a major determinant of all-cause mortality and reduced lung function post-TB is likely to contribute to the reported reduced survival after TB treatment.^[31]^ Reduced spirometry is the most reported post-TB complication and most participants in this present study had severe lung function impairment.^[32–33]^ The severity of illness in these participants referred for specialist care compared with previous screening studies could be explained by the long duration of illness or more severe disease warranting referral. Although obstructive spirometry and COPD have received the greatest attention as TB treatment sequelae, the mixed pattern, as we have reported in the present study, is the most common impairment.^[34]^ The mechanism underlining post-TB lung function is complex and may contribute to the mixed pattern. In addition to airway disease and obstruction, coexisting structural damage with cavitation, bronchiectasis, lung collapse, destroyed lungs and aspergilloma are likely to lead to adjunct restrictive impairment.^[9]^ Therefore, identification and treatment of structural lung damage post-TB is a possible strategy that could improve lung function. Furthermore, localised resection of a lung is an under-utilised option that may provide long-term benefits when performed early. However, early identification and intervention relies on a clear follow-up pathway after TB treatment, but this practice is currently lacking. The long median duration between TB treatment and presentation for post-TB care at our clinic gives credence to the current gaps in follow-up care.

Although residual structural lung damage following TB treatment increases the risk of *Mycobacterium tuberculosis* reinfection, nonetheless, repeated TB treatment is commonly given despite negative sputum tests.^[35]^ The inference is that of low awareness and recognition of post-TB lung disease by healthcare providers, which is enabled by the absence of national TB guidelines for diagnosis. Repeated TB treatment is not innocuous and should be avoided in the absence of active TB. Emerging evidence demonstrates that TB drugs could cause profound and long-lasting dysbiosis of the gut microbiota and risk of bacteria developing resistance, increased susceptibility to pathogens, and altered immune tolerance.^[36]^

### Study limitations

The relatively small sample size that precluded multivariate analyses limits the strength of the associations we have described, making them only exploratory. There is also significant selection bias since the patients that were recruited into this present study were from a specialist post-TB clinic. This means that our findings are likely to represent severe post-TB lung disease and not the complete disease spectrum, which may limit extrapolation to other populations. The GLI African American reference equation we used to classify and grade spirometry impairment has not been validated in the Nigerian population and may have overdiagnosed or underdiagnosed spirometry impairments. Nonetheless, we have provided data on the clinical profile of post-TB lung disease in Nigeria, which has brought to the fore the high burden of disease and the need for improved awareness and recognition, early diagnosis, and the quest for appropriate treatment

## Conclusion

We demonstrated that post-TB lung disease is associated with a high symptom burden, severe spirometry impairment and poor HRQoL. Increasing dyspnoea, sputum production, cough, and weight loss are associated with poor HRQoL and could be targets for intervention. This present study underscores the need for increased recognition and development of guidelines for the diagnosis and treatment of post-TB lung disease and calls for further research to explore preventive strategies.
